# Single point mutation in Rabenosyn-5 in a female with intractable seizures and evidence of defective endocytotic trafficking

**DOI:** 10.1186/s13023-014-0141-5

**Published:** 2014-09-20

**Authors:** Sylvia Stockler, Silvia Corvera, David Lambright, Kevin Fogarty, Ekaterina Nosova, Deborah Leonard, Robert Steinfeld, Cameron Ackerley, Casper Shyr, Nicolas Au, Kathrin Selby, Margot van Allen, Hilary Vallance, Ron Wevers, David Watkins, David Rosenblatt, Colin J Ross, Elizabeth Conibear, Wyeth Wasserman, Clara van Karnebeek

**Affiliations:** Division of Biochemical Diseases, B.C. Children’s Hospital, Vancouver, Canada; Department of Pediatrics, University of British Columbia, Vancouver, Canada; Treatable Intellectual Disability Endeavour in British Columbia, B.C. Children’s Hospital, Vancouver, Canada; Center for Molecular Medicine and Therapeutics, Child and Family Research Institute, Vancouver, Canada; Department of Medical Genetics, University of British Columbia, Vancouver, Canada; Department of Pathology & Laboratory Medicine, University of British Columbia, Vancouver, Canada; Division of Pediatric Neurology, B.C. Children’s Hospital, Vancouver, Canada; Biochemical Genetics Laboratory, Children’s and Women’s Hospital, Vancouver, Canada; Program in Molecular Medicine, University of Massachusetts Medical School, Worcester, MA USA; Biomedical Imaging Group, University of Massachusetts Medical School, Worcester, MA USA; Department of Pediatrics, University Medical Center Goettingen, Goettingen, Germany; Division of Pathology, Hospital for Sick Children, Toronto, Ontario Canada; Department of Laboratory Medicine, Radboud University Medical Centre, Nijmegen, The Netherlands; Department of Human Genetics, McGill University, Montreal, Québec, Canada

**Keywords:** Internalization, Receptor endocytosis, Recycling, FYVE domain, Rab GTPase, Epilepsy, Cathepsin D, Vitamin B12, Inborn error of metabolism

## Abstract

**Background:**

We report a 6.5 year-old female with a homozygous missense mutation in *ZFYVE20*, encoding Rabenosyn-5 (Rbsn-5), a highly conserved multi-domain protein implicated in receptor-mediated endocytosis. The clinical presentation includes intractable seizures, developmental delay, microcephaly, dysostosis, osteopenia, craniofacial dysmorphism, macrocytosis and megaloblastoid erythropoiesis. Biochemical findings include transient cobalamin deficiency, severe hypertriglyceridemia upon ketogenic diet, microalbuminuria and partial cathepsin D deficiency.

**Methods and results:**

Whole exome sequencing followed by Sanger sequencing confirmed a rare (frequency:0.003987) homozygous missense mutation, g.15,116,371 G > A (c.1273G > A), in *ZFYVE20* resulting in an amino acid change from Glycine to Arginine at position 425 of the Rbsn protein (p.Gly425Arg), as the only mutation segregating with disease in the family. Studies in fibroblasts revealed expression and localization of Rbsn-5G425R in wild-type manner, but a 50% decrease in transferrin accumulation, which is corrected by wild-type allele transfection. Furthermore, the patient’s fibroblasts displayed an impaired proliferation rate, cytoskeletal and lysosomal abnormalities.

**Conclusion:**

These results are consistent with a functional defect in the early endocytic pathway resulting from mutation in Rbsn-5, which secondarily disrupts multiple cellular functions dependent on endocytosis, leading to a severe multi-organ disorder.

## Introduction

As of 2014, an estimated 1,800 Mendelian conditions remain to be identified (http://www.omim.org/statistics/entry). We recently have established a research program (www.tidebc.org) using whole exome sequencing (WES) in patients with unexplained metabolic phenotypes to diagnose/discover inborn errors of metabolism as a potential cause of their intellectual disability and/or developmental delay [1]. Here we report such a case, in which a mutation in the gene *ZFYVE20*, encoding the protein Rabenosyn-5 (Rbsn-5), is the likely cause of a complex condition in a female child characterized by developmental delay, intractable epileptic encephalopathy, and a pleiotropic clinical and biochemical phenotype.

Rbsn-5 is a large, evolutionarily conserved multidomain protein expressed ubiquitously in mammalian cells and implicated in receptor-mediated endocytosis [2-5]. The specific role of Rbsn-5 is to regulate the intracellular route of internalized receptors, facilitating their recycling to the plasma membrane [5-7]. By controlling the rate of recycling of numerous receptors, Rbsn-5 affects critical cellular functions, amongst which are the absorption and cellular uptake of high molecular weight nutrients, the signaling capacity of growth factor and neurotransmitter receptors, and the ability of integrins to control cellular motility. At the molecular level, Rbsn-5 controls receptor recycling [5,7] through direct interactions with regulatory proteins and lipids, which include the endocytic GTPases Rab4 and Rab5, and phosphatidylinositol-3 phosphate, the product of the class III PI-3 kinase Vps34 [6,8-10]. Thus, mutations that cause structural alterations in specific regions of Rbsn-5 are likely to disrupt the balance of interactions with one or more of these regulatory factors, and lead to widespread abnormalities in receptor recycling dynamics.

Transferrin (Tf) is frequently used to monitor endocytosis and recycling, due to the abundance of transferrin receptors in primary and cultured cells, as well as the property of Tf to become internalized and quantitatively recycled. Rbsn-5 depletion in cultured mammalian cells impairs Tf trafficking [5-7] by interfering with recycling, and can lead to decreased receptor levels [5]. High-resolution live cell imaging studies have shown that Rbsn-5 is localized to endosomes to which Tf is delivered immediately following its internalization from clathrin-coated pits at the plasma membrane [5].

We have used this approach to determine how the mutation in Rbsn-5 in this patient impairs endocytosis and recycling. Additional biochemical and morphological approaches have been used to further understand the pleiotropic cellular consequences of this disruption. Possible mechanisms by which these defects culminate in the overall clinical presentation in this patient are discussed.

## Methods

This study was initiated as part of the Treatable Intellectual Disability Endeavor in British Columbia and approved by the institutional review boards of BC Children’s Hospital and the University of British Columbia (CW12-0019/H12-00067). Parents provided written informed consent.

### Whole exome sequencing

Index and unaffected parental sequencing was conducted using Agilent SureSelect kit and Illumina HiSeq 2000 (Perkin-Elmer, Santa Clara, California, USA) (REB Approval CW12-0019 / H12-00067) Approximately 50.2 million 100 bp pair-end reads were generated per participant. A combination of Bowtie, BWA and GSNAP was used to map the reads to hg19 reference genome, and Samtools was used to call the variations relative to the reference. On average, 99% of the observed variations were classified as common variants based on a reported allele frequency in dbSNP (version 135). The remaining set of rare or novel variants (defined as <0.01 minor allele frequency) was assessed for potential to disrupt protein function using the Sift and PolyPhen2 software systems, and was also screened under a series of genetic models with a focus on Mendelian recessive modes of inheritance.

### PCR

Exome sequencing results of the mutations in *ZFYVE20* were validated in the index and unaffected parents (with targeted mutation analysis in the unaffected siblings), Sanger DNA sequencing using standard PCR conditions with an annealing temp of 59°C. (primer 1: GGGTCTGAGTCCTCACTCTGC, primer 2: TGTCACTGGCACAGGGATAG).

### Cell culture and transfection

CB and PA cells were maintained in MEM 2% FBS. Cells were transfected using electroporation, plated on glass coverslips (Thomas Scientific 25 circle #1.5), and grown for 48 hours. Live imaging was done in KRH buffer (125 M NaCl, 5 mM KCl, 1.3 mM CaCl_2_, 1.2 mM MgSO_4_, 25 mM HEPES, 2.5% BSA and 2 mM sodium pyruvate) pH 7.4.

### TIRF/Epi-fluorescence structure-illumination microscope (TESM) optical system

A custom-built microscope system, TESM, simultaneously combines Total Internal Reflection Fluorescence and wide-field epifluorescence modes and incorporates structured illumination in the epi mode for fast optical sectioning and enhanced spatial resolution. Further details as well as the TESM acquisition system is described previously [5]. For quantification, image sets were convolved with a difference of Gaussians (DOG) filter consisting of 1) a small, two dimensional, Gaussian spot with unit area (sigma = 150 nm) that acted as a vesicle matched detector, i.e. an approximation to a near-diffraction limited spot, and 2) a larger, inverted, two dimensional Gaussian (sigma = 300 nm) with negative unit area that estimated and subtracted the local background. The Gaussian smoothed images were visually thresholded (global threshold) to select for pixels belonging to objects (e.g. vesicles) and eliminate areas devoid of signal (but containing noise). The total intensity per cell over time was recorded.

### Reagents

The TagRFP-T expression vector was constructed as described [11]. The cDNA clone 40034008 for human Rabenosyn-5 was obtained from American Tissue Culture Collection (Manassas, VA) and was cloned in frame with TagRFP-T at the N terminus of the protein using standard techniques. Polyclonal EEA1 and Rbsn-5 antibodies have been described [5]. Unconjugated and DyLight-conjugated human transferrin were obtained from Jackson Immunochemicals.

### Electron microscopy

Fibroblasts were fixed and postfixed in phosphate buffered 2.5% glutaraldehyde and osmium tetroxide respectively, dehydrated in an ascending series of acetone and infiltrated and embedded in epon araldite, and ultrathin sections were prepared and mounted on grids prior to examination in the electron microscope.

### Cathepsin D analysis

Goat anti-human CatD antibody was used to visualize the 53-kDa proenzyme precursor (preCatD) and the processed 33 kDa heavy chain (CatD) of cathepsin D by western blotting. Blots were stained with Lumi Light Western blotting substrate (Roche Diagnostics, Mannheim, Germany) to visualize the 53-kDa proenzyme precursor (preCatD) and the processed 33 kDa heavy chain (CatD) of cathepsin D by chemiluminescence [12].

## Results

### History and physical exam

This 6.5 year-old girl was born as the second of 3 sisters to healthy non-consanguineous Caucasian parents after an uneventful pregnancy. She developed pharmaco-resistant infantile spasms at age 5 months, which improved upon ketogenic diet (KD) started at age 14 months. Severe hypertriglyceridemia (plasma triglycerides: 90 mmol/L, normal 0.36-1.31) observed at age 50 months was reversible by reduction of natural fat in the KD, and partial replacement with medium chain triglycerides. Her cranial 1.5 T MRI, at age 40 months showed moderate enlargement of the 3^rd^ ventricle, normal myelination and cortical architecture.

**Figure 1:**
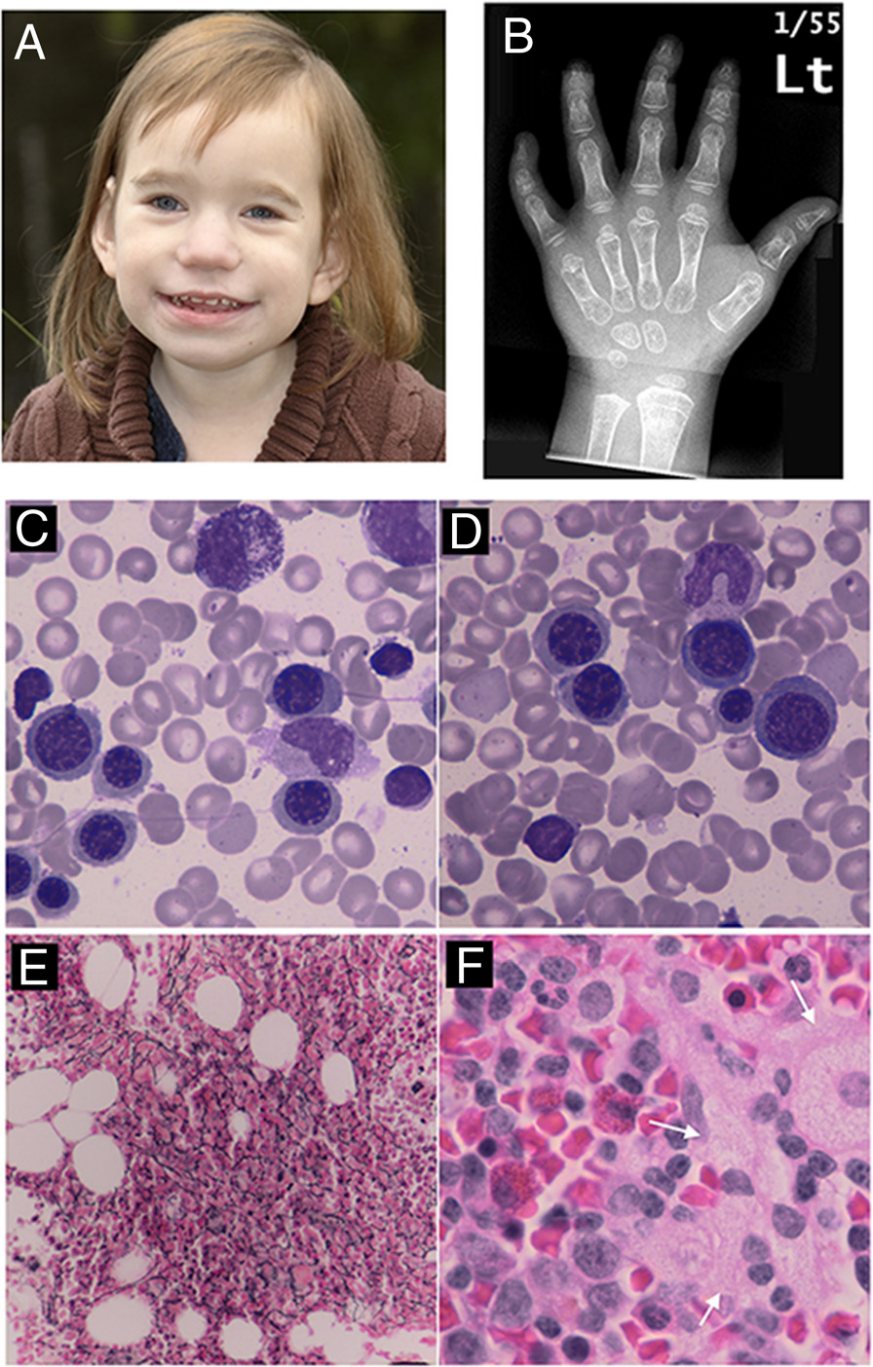
**Clinical features of a girl with a point mutation in Rabenosyn-5. A**: Facial features showing midfacial bone hypoplasia, deep set eyes with a hooded appearance, a fullness in the nasal bridge, a short nose and a large mouth with small teeth and tongue protrusion. **B**: PA view of right hand showing marked under mineralization, coned epiphyses, under tubulated long bones, and relatively short digits particularly involving the middle phalanges of digit 2 and digit 5. Delayed skeletal maturation at approximately 2 years 6 months for a chronological age of 6 years 2 months and a single standard deviation of 10 months. **C**-**F**: Bone marrow aspirates showing megaloblastoid erythropoiesis **(C and** **D)**, occasional intercytoplasmic bridging **(C)**, mild increase in reticulin fibrosis throughout most of the bone marrow **(E)**. Foamy macrophages most notably in the bone marrow biopsy specimen **(F)**.

At 6.5 y she is clinically seizure free upon the modified KD and four anticonvulsive drugs (Valproic acid, Phenobarbital, Levetiracetam, Lamotrigine). Her biparietal head circumference is 46 cm (below the 0.1^st^ percentile); weight and height at the 3^rd^ and 0.1^st^ percentile. She is hypotonic, able to sit without support, but unable to stand or walk. She has a happy, friendly demeanor, is non-verbal and unable to feed herself and not toilet-trained. She has thin, whispy hair and dysmorphic facial features (Figure 1A).

A skeletal survey at chronological age 6 years revealed a moderate osteopenia involving the pelvis and long bones of both upper and lower limbs with evidence of undertubulation and hypoplasia of the epiphyses around the knee joint and bilateral coxa valga. Particularly the long bones of the right hand appear undertubulated and osteopenic. (Figure 1B) with delayed appearance of the right carpal bones (approximate bone age 2.5 years).

### Clinical biochemistry

At 14 months of age (prior to start of the ketogenic diet), biochemical genetic assessment revealed repeatedly normal results for blood lactate and ammonia levels, plasma and CSF aminoacids, plasma very long chain fatty acids, pipecolic acid, and transferrin phenotyping, urine excretion of purines and pyrimidines, creatine, guanidinoacetate, glycosaminoglycans, oligosaccharides and α-aminoadipic acid semialdehyde. Urinary organic acid analysis revealed high urine methylmalonic acid concentration (40 mmol/mol creatinine, normal < 5.13) along with mildly elevated plasma homocysteine (13.7 μmol/L, normal <7) and low cobalamin (vitamin B12) levels (66 and 60 pmol/L, deficient range < 107). The child was breastfed until age 14 months when she started the ketogenic diet. The mother had been on a normal, vitamin B12 containing diet throughout pregnancy and the time of breastfeeding. At the time vitamin B12 deficiency was diagnosed in her child, her plasma vitamin B12 levels were within non-deficient range (178 pmol/L, normal 156–698), her plasma homocysteine level was 13 μmol/L (normal 6–12.8), her CBC, urine organic acid profile and blood acylcarnitine profile were normal. Cobalamin levels in the child increased significantly (up to 1107 pmol/L) after intramuscular injection of vitamin B12 (cyanocobalamin 1000 μg) on 10 subsequent days, and remained within non-deficient range (471 pmol/L, non deficient range: 133–675) upon continuous oral supplementation of cyanocobalamin (250 μg per day) when measured at 26 months of age. Homocysteine remained normal when measured at various occasions (3.1, 5.0, 2.5 μmol/L), but urine and serum MMA levels remained mildly elevated when measured at 29 and 40 months of age (urine: 31 and 32 mmol/mol creatinine, normal < 5.13; serum: 0.21 at 40 months, normal 0.018-0.150).

Defects of cobalamin absorption, transport and intracellular metabolism were excluded by respective tests in blood and cultured fibroblasts (Table 1). Also detected were microalbuminuria (microalbumin/creatinine ratio = 15.6 - 26.8, normal <2.7) and high urinary Ca excretion (calcium/creatine ratio = 3.73, normal <1.1) in the presence of normal plasma 25 OH Vitamin D, calcium, phosphorus and alkaline phosphatase levels.

**Table 1:** **Studies of cobalamin absorption, transport, cellular uptake and intracellular metabolism in a patient with a homozygous G425R mutation in** ***ZFYVE20***

Intrinsic Factor antibodies	Negative antibodies
Parietal Cell antibodies	
Gastric Intrinsic Factor Protein *(GIF)*	No pathogenic mutations on WES (coverage × 30, last 5 exons of AMN × 3–5)
Cubilin *(CUB)*	
Megalin *(LRP2)*	
Amnioless *(AMN)*	
Total Haptocorrin (TCN1)	592 pmol/L (normal 240–680)
Plasma Total Transcobalamin (TCNII)*	1520 pmol/L (normal 500–1.500)
Plasma Holotranscobalamin*	182 pmol/L (normal 40–160)
Transcobalamin Receptor related genes *(TCbR, CD320, TCN1, TCN2)*	Absence of (262_264delGAG and 297delA) by targeted mutation analysis [17]
	No pathogenic mutations by WES (coverage > × 20)
[^14^C]-propionate incorporation (f)	11.0 nmol/mg protein/18 h (normal 10.8 ± 3.7 nmol/mg protein/18 h)
[^14^C]-methylene tetrahydrofolate incorporation (f)	250 pmol/mg protein/18 h (normal 225 ± 165 pmol/mg protein/18 h)
[^57^Co]-cyanocobalamin uptake (f)	16.9 pg/10^6^ cells (normal 13.2 ± 4.8 pg/10^6^)
[^57^Co]-cyanocobalamin conversion to adenosylcobalamin (f)	5.7. % of intracellular cobalamin (normal 15.3 ± 4.2)
[^57^Co]-cyanocobalamin coversion to methylcobalamin (f)	53.0% (normal 58.0 ± 6.7)

(f) = fibroblasts; *by courtesy Ebba Nexo, Arhus, DK.

### Clinical hematologic findings

Red blood cell macrocytosis (range: 95.5-104.6 fl; mean: 98.8; n = 21; normal 75–87) was noted prior to initiation of the ketogenic diet and persisted throughout the entire observation period. Transient neutropenia occurred between age 40 and 44 months (range: 0.7-1.3 × 10^9^/L, mean: 0.9, n = 4; normal: 1.5-8.5). A bone marrow aspirate and biopsy at age 44 months showed normocellularity for age with a normal myeloid to erythroid ratio (2:1) (Figure 1 C-F). A mild increase in reticulin fibrosis (2+ out of 4) but no collagen deposition were present. No significant dysplastic features were seen in the granulocytic and megakaryocytic lineages. Foamy macrophages were attributed to the marked hyperlipidemia present at the time of bone marrow sampling. Peripheral blood morphology at the time of bone marrow biopsy showed increased target cells and stomatocytes. Erythropoiesis showed megaloblastoid change with some erythroblasts showing features approaching a true megalosblastic state, however no neutrophil hypersegmentation nor giant myelocytes or metamyelocytes were present and iron stains did not show ring sideroblasts. Cytogenetic studies were unremarkable at the time. Plasma copper, zinc and folate, erythrocyte folate, serum iron and ferritin and transferrin saturation were normal on repeated occasions.

### Genetic analyses

The patients karyotype and chromosomal microarray analysis (AffymetrixCytoscan® HD) were unremarkable; homozygosity analysis did not reveal evidence of consanguinity or uniparental disomy. WES was performed for the index and her unaffected parents. Rare variants were assessed for their potential to disrupt protein function and screened under a series of genetic models—primarily the Mendelian recessive mode of inheritance given the rarity of the phenotype and the pattern of inheritance of most IEMs.

**Figure 2:**
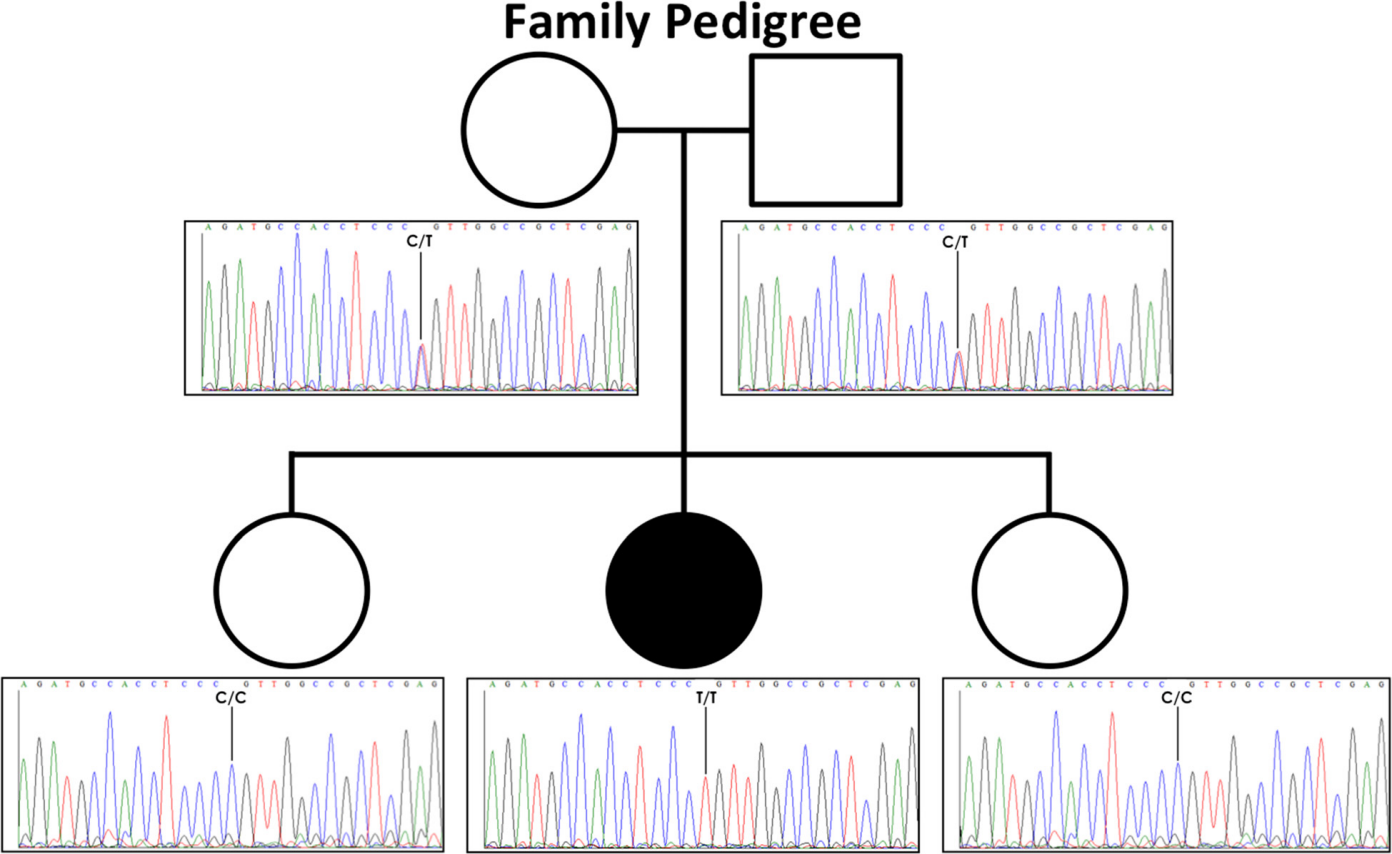
**Pedigree of affected case.** Circles represent females, square represents male, black symbol represent affected case.

Approximately 99% of the observed variations were classified as common *(results not shown*). Two rare (defined as <0.01 minor allele frequency) candidate variants fit the compound heterozygous model autosomal recessive model of homozygous WES revealed a total of 5 genes harboring compound heterozygous variants and 3 genes with homozygous variants (*SLC41A3*, *CLIP*, *ZFYVE20)* Only the homozygous missense variation (g. 15,116,371 G > A) in *ZFYVE20*, resulting in a glycine to arginine substitution at position 425 (p.Gly425Arg), segregated with disease in the family as confirmed by Sanger sequencing (Figure 2).

### Cellular endocytosis and recycling analysis

**Figure 3:**
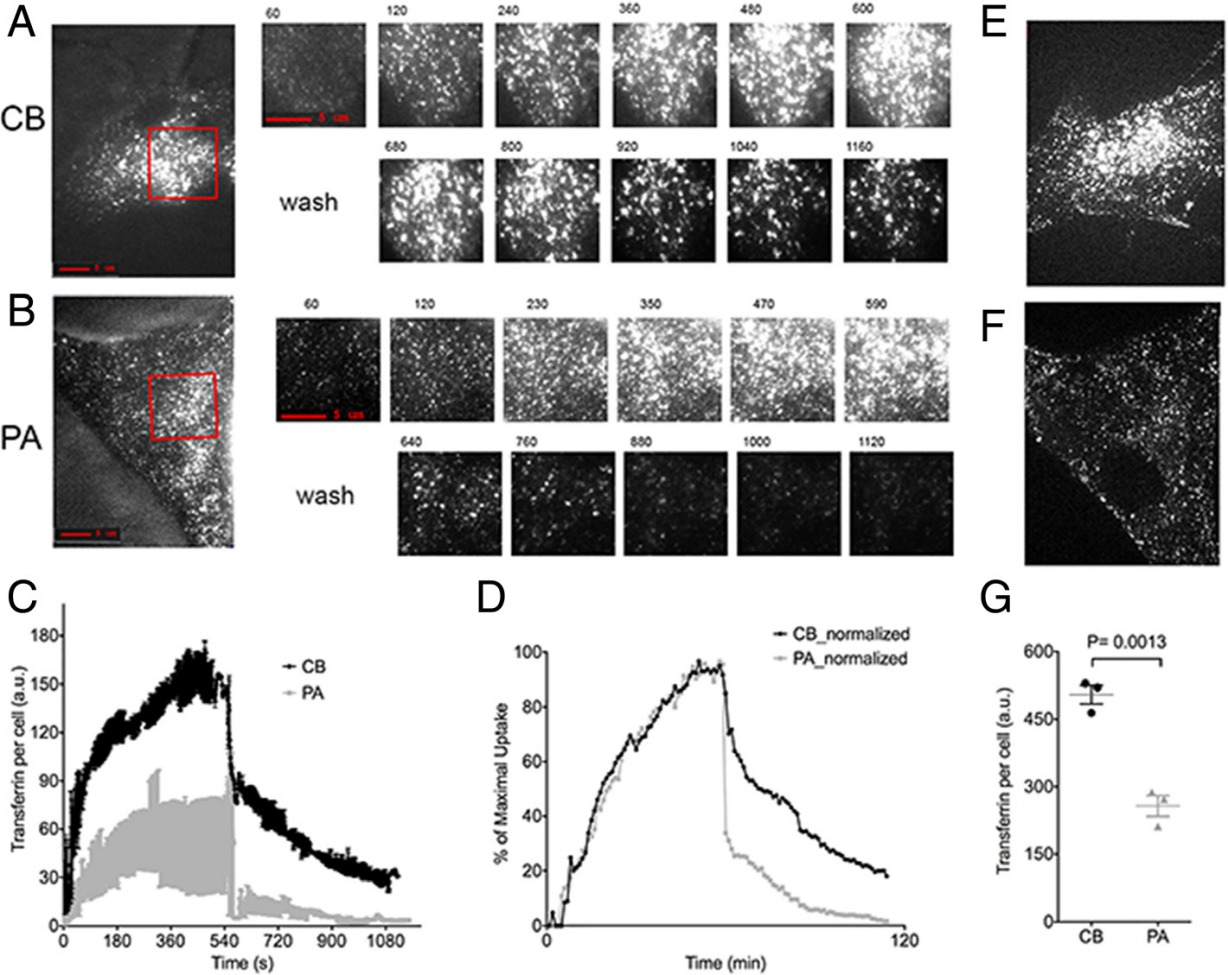
**Dynamics of transferrin association and dissociation from human fibroblasts.** TIRF images of the entire cell from control **(A)** or affected **(B)** individual at 300 seconds following addition of fluorescent transferrin. Red rectangles represent region depicted in the time series shown to the right. Transferrin was removed from the medium at t = 600. **C**. Quantification of the amount of transferrin in the TIRF image of the entire cell, plotted as the mean and SEM of each time point from three individual cells. **D**. The mean from the data in C was recalculated as a percent of maximal signal. Structured light epifluorescence image stacks from control **(E)** or affected **(F)** individual taken at t = 600, projected into a single plane. **G**. Quantification of signal in the structured light images from 3 individual cells. Statistical significance was calculated by 2-tailed Student t-test. Similar experiments were conducted with three independent passages, with similar results.

The uptake and recycling rates of transferrin (Tf) in patient (PA) and age and passage matched control (CB) fibroblasts were studied using described methods [5,13]. In brief, cells were imaged by total internal reflection fluoresce microscopy (TIRF) at 1 frame/sec continuously for 20 minutes, during which Alexa-488-Tf was added to the medium, maintained for 10 minutes and then removed. Raw TIRF images obtained over time from control (Figure 3A) or patient (Figure 3B) single cells, together with mean and SEM from three independent cells are illustrated in Figure 3C. Data normalized to the maximal uptake recorded in each cell is shown (Figure 3D).

Tf associated rapidly and saturably with both PA and CB cells, displaying kinetic constants consistent with binding to the transferrin receptor [13-16], and similar to those previously determined in other mammalian cell types. However, in PA cells Tf saturated at a lower maximal rate (Figure 3C), and displayed a significantly faster rate of recycling compared to CB cells (Figure 3C,D,G). The accumulation of Tf in the three dimensional volume of the cell was visualized immediately before the removal of extracellular Tf, by collecting 10 image stacks using structured light illumination. Accumulation of Tf in PA cells was only 50% of that observed in CB cells (Figure 3E,F). Results demonstrate a defect in the early endocytic pathway with enhanced recycling rate and decreased steady-state accumulation of ligand in cells harboring the p.G425R mutation.

**Figure 4:**
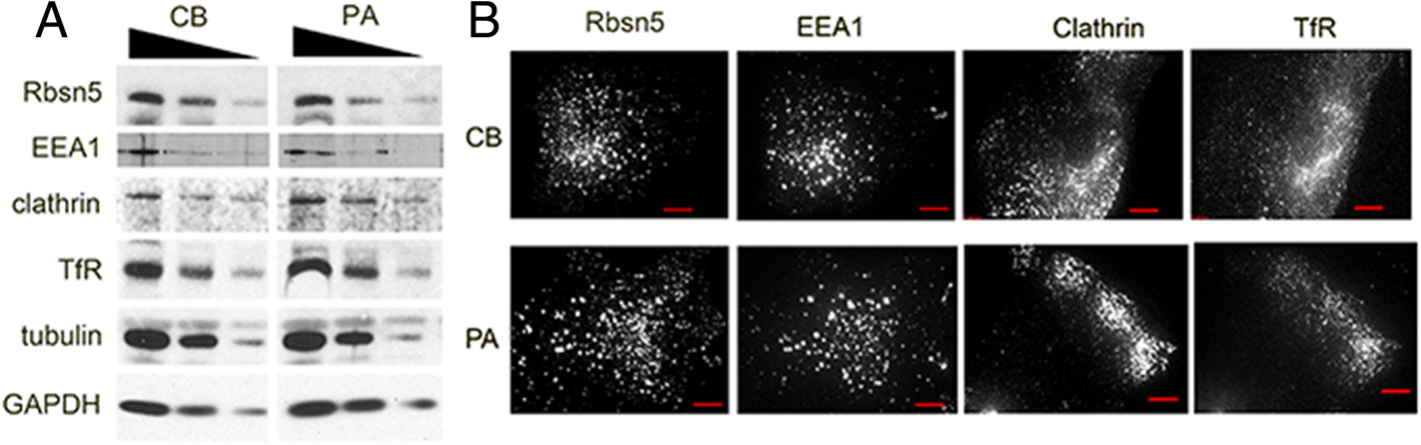
**Western blotting and subcellular distribution of endocytic pathway proteins. A**. Western blotting: Cells from control (CB) or affected (PA) individual were grown to 80% confluence, and whole cell lysates obtained. Serial dilutions of equal total protein concentrations of the cell extracts were processed to enable quantification, as indicated. **B**. Subcellular distribution: Cells from control (CB) or affected (PA) individual were grown on glass coverslips, fixed, permeabilized and stained with antibodies to the proteins indicated above each panel. Shown in black and white are structured light epifluorescence image stacks from representative cells, projected into a single plane.

Western blotting did not show significant differences between PA and CB total cellular levels of Rbsn-5 and other key proteins within the early endocytic pathway (Figure 4A), ruling out the possibility that the p.G425R mutation might result in loss of protein stability and enhanced degradation. Moreover, no consistent difference in the total level of the transferrin receptor (TfR) was noted, suggesting that the alterations in Tf endocytosis in PA cells are not simply a consequence of lower receptor levels. Immunofluorescence staining with antibodies to Rbsn-5, EEA1, clathrin and TfR showed similar distribution in CB and PA cells and comparable to that previously described for Cos-1 cells (Figure 4B).

**Figure 5:**
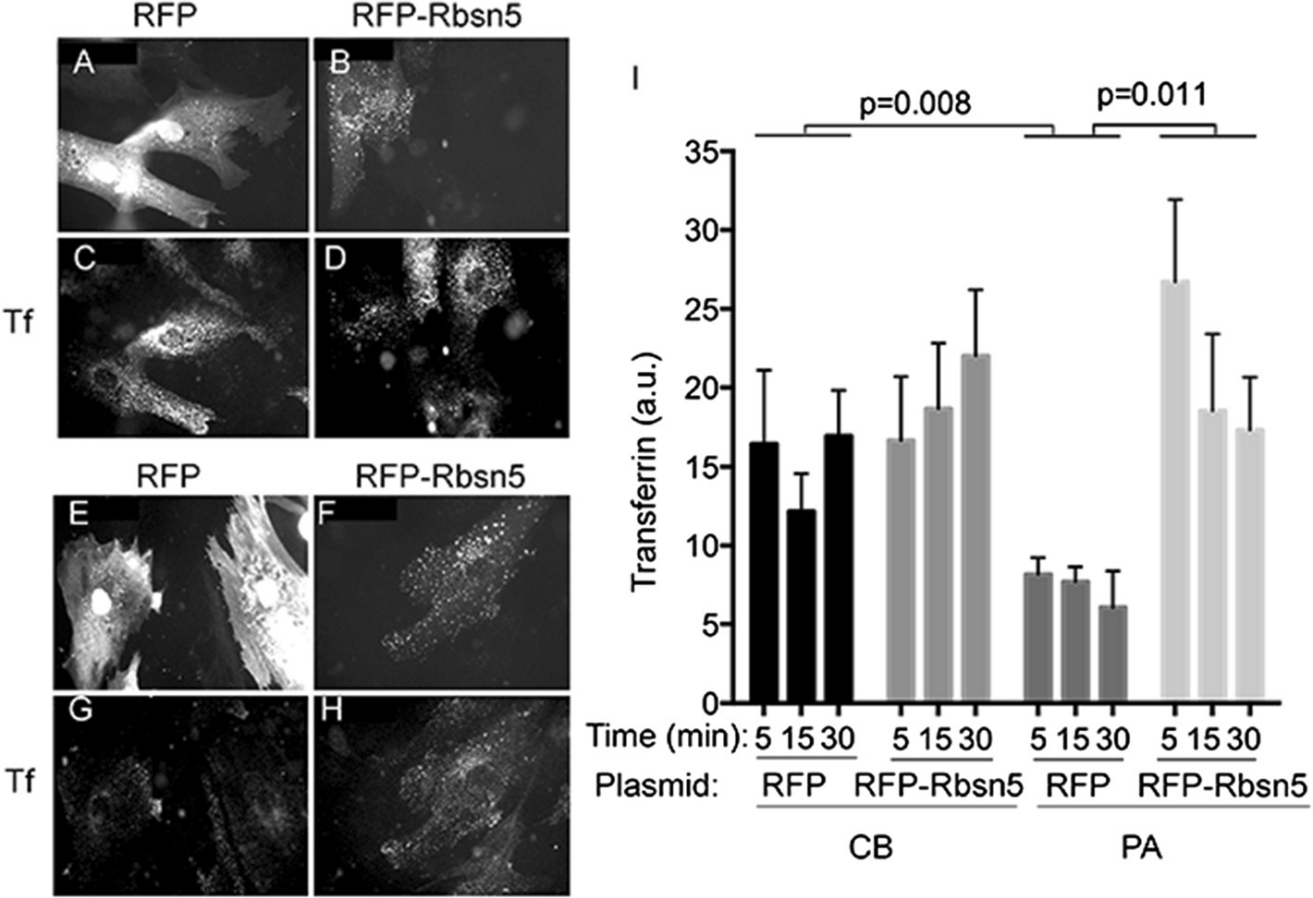
**Rescue of Tf accumulation by wild-type Rbsn-5.** Cells from CB **(A-D)** or PA **(E-H)** were transfected with plasmids encoding RFP **(A,C,E,G)** or RFP-Rbsn5 **(B,D,F,H)**. After 48 h, cells were exposed to Alexa-488-Transferrin for 5–30 min, washed briefly and fixed. Representative images of RFP **(A,E)** or RFP-Rbsn5 **(B,F)** and corresponding Tf images **(C,G and** **D,H)**. Light intensity of Tf signal per cell was quantified **(I)**. Plotted are means and SEM of 50–100 cells from 4 independent experiments. Statistical significance was calculated using 2-tailed paired Student t-test, where the means of each time point are paired as indicated.

Finally, we investigated restoration of function transfecting PA and BC cells by electroporation with either a control plasmid encoding soluble RFP or RFP-Rbsn-5. After 48 h, cells were exposed to Alexa488-Tf for the indicated time (Figure 5) and accumulation per cell was measured. CB fibroblasts expressing RFP (Figure 5A,C) or RFP-Rbsn-5 (Figure 5B,D) accumulated a similar amount of Tf over different time points analyzed (Figure 5C,D and I). In contrast, Tf uptake into PA cells was lower than that seen in CB cells, and significantly increased upon expression of RFP-Rbsn-5 (Figure 5G,H and I). Experiments suggest that the observed defect in transferrin trafficking is attributable to the Rbsn-5 G425R mutation.

**Figure 6:**
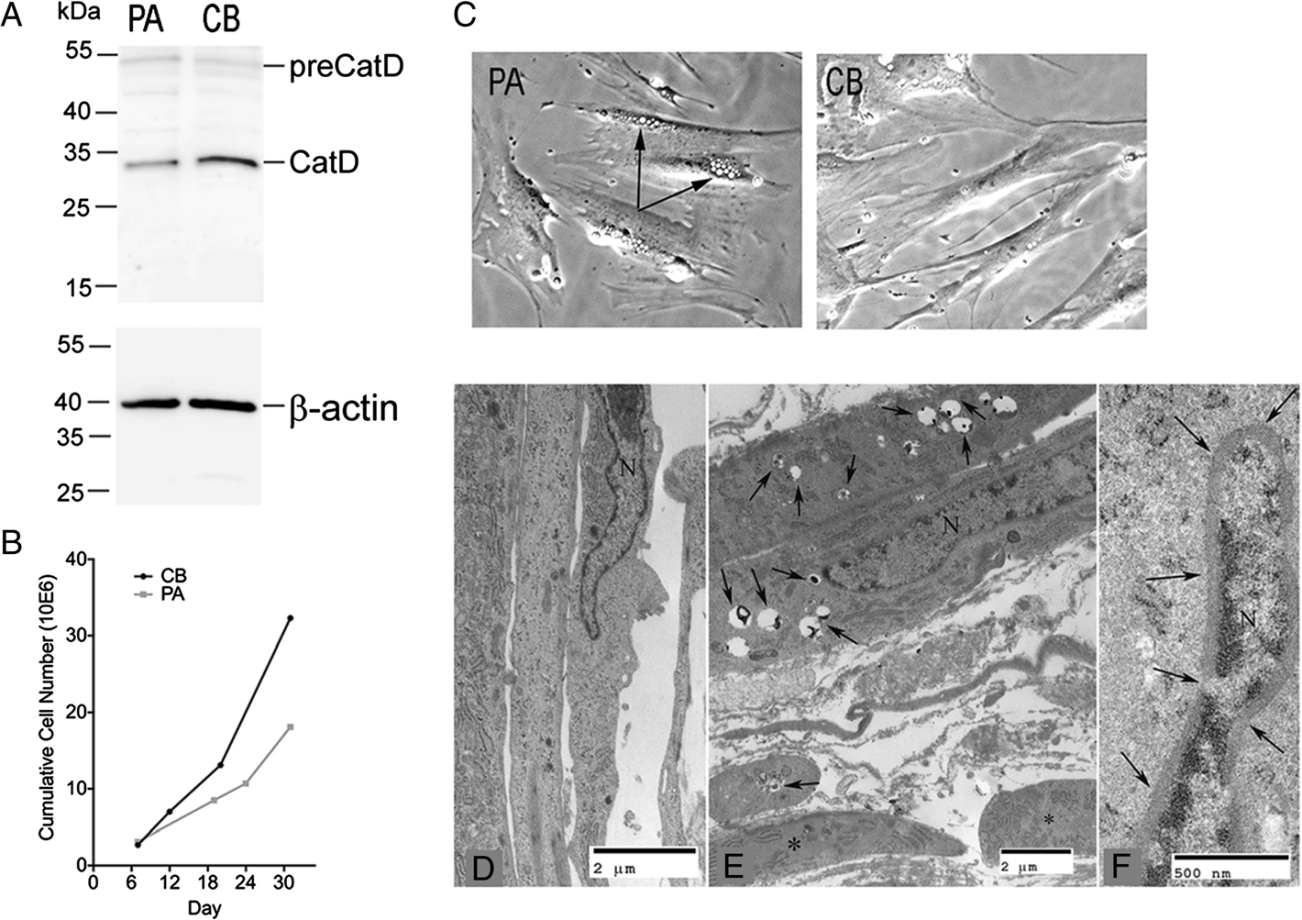
**Additional findings in fibroblasts A.** Western blot of extracts from cells from control (CB) or affected (PA) individual with goat anti-human CatD antibody, indicating 53-kDa proenzyme precursor (preCatD) and the processed 33 kDa heavy chain (CatD) of cathepsin-D. Detection of β-actin demonstrated similar amounts of protein loading for patient and control lysates. **B**. Cumulative cell numbers calculated at each passage over a 30 day period. **C**. Phase images of CB (left) and PA (right) fibrobasts. Arrows indicate translucent vacuoles present in many of the PA cells. Electron micrographs of normal **(D)** and patient **(E)** fibroblasts. **F**. Nucleus (N) from patient’s fibroblast surrounded by a dense band of filamentous material.

We then tested whether the effect of the Rbsn-5 p.G425R mutation might be restricted to Tf recycling kinetics, or as expected, would affect the activities of other receptors. Depletion of Rbsn-5 causes altered subcellular distribution of mannose-6-phosphate receptors affecting the biosynthetic transport of cathepsin D from Golgi to the lysosomal lumen [17]. Consistent with this function, we found a reduction of cathepsin D activity to 35% of the one measured in control fibroblasts (404 ± 53 versus 1158 ± 178 nmol/h × mg, n = 3). In Western blotting, the intensity of precursor cathepsin D was slightly higher in the patient fibroblast, in contrast the intensity of 33 kDa heavy chain of the mature cathepsin D was diminished to 42% of the control (Figure 6A), indicating compromised cathepsin D processing to mature, proteolytic active cathepsin D. Targeted WES analysis of *CTSD* (with ≥25X coverage of all exons) did not identify any rare, damaging variants.

To further assess the consequences of impaired trafficking, growth rates and subcellular morphology of the PA cells were analyzed. A slower proliferation rate was found (Figure 6B), as well as the presence of translucent vacuoles in the perinuclear region (Figure 6C). These vacuoles were negative for Tf even after prolonged uptake, and negative for lysotracker staining (not shown). The presence of vacuoles was not mitigated by transient expression of Rbsn-5, and thus may be the result of persistent changes caused by endocytic pathway abnormalities, which are not reversible in a short-term experiment. Electron micrographs of the patient’s fibroblasts (Figure 6D-F) showed an excess of intermediate filaments in either a perinuclear band, associated with the plasmalemma, or throughout the cytoplasm, as well as lysosomes containing small granules of electron opaque material. Cytoplasmic organelles were sparse when compared to fibroblasts derived from normal individuals.

## Discussion

Here we report a complex clinical phenotype associated with a mutation in exon 14 of *ZFYVE20*, leading to mutation of glycine 425 to arginine in Rbsn-5, which appears to affect the dynamics of receptor trafficking in the endocytic pathway. To our knowledge this is the first report of mutations in this highly conserved multidomain protein, and the complex ensuing clinical phenotype is consistent with the fundamental role of endocytosis and recycling in mammalian cell function, and with the critical role that Rbsn-5 plays in this multistep process.

**Figure 7:**
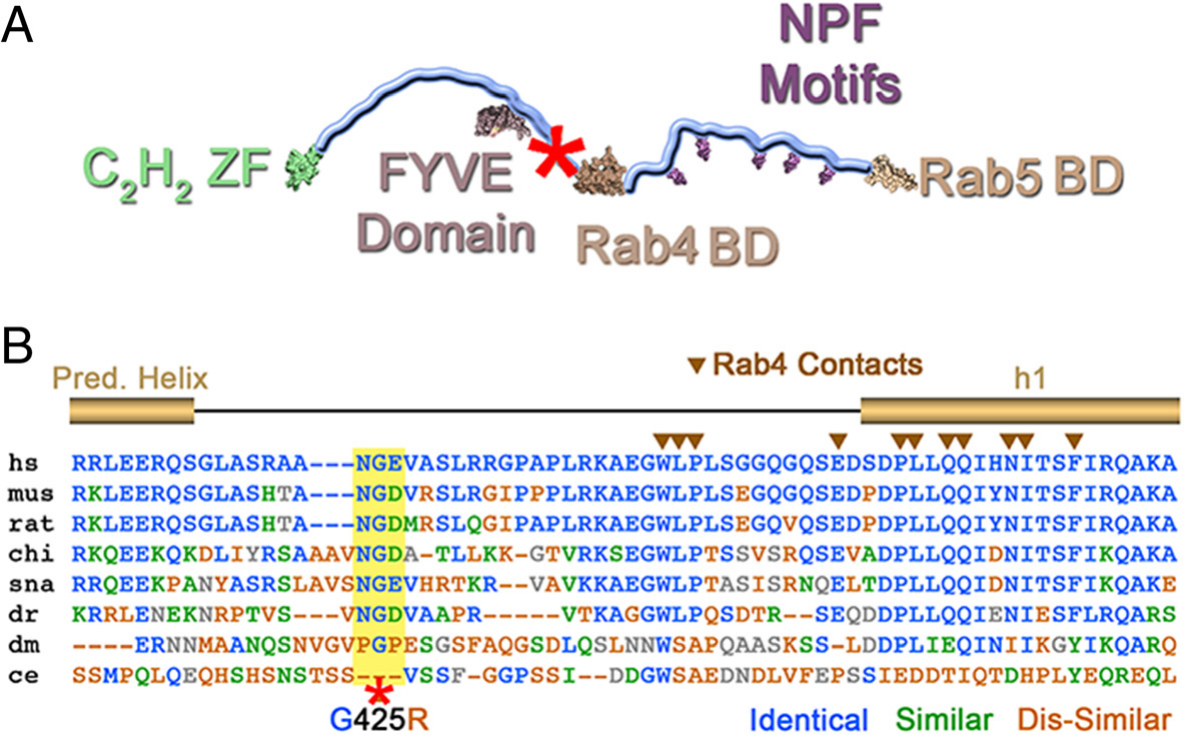
**Position of G425R mutation in the context of full-length Rbsn-5. A**. Diagram of Rabenosyn-5 with functional domains indicated. Red asterisk indicates position of mutation. **B**. Amino acid conservation among species in the region of the mutation.

The location of this mutation is in the central residue in an NG(D/E) motif that stands out as an island of conservation within an otherwise variable segment in higher metazoans (mammals, birds, fish, reptiles) (Figure 7). We are currently investigating the effect of this mutation on the tertiary structure of the protein using X-ray crystallography and small angle X-ray scattering. Mutation to arginine as found in this patient, represents a major, non-conservative change that could result in loss of function with respect to flexibility, structural organization, and/or intermolecular interactions, in addition to potential effects on membrane targeting, stability, solubility, or oligomeric state. These alterations are likely to affect the function of Rbsn-5 to generally control receptor trafficking, which in turn would affect the levels and activity of multiple receptor species in multiple cell types, thus underlying multi-organ level abnormalities. Consistent with this possibility, we find evidence for enhanced receptor recycling when directly visualizing Tf endocytosis, as well as reduced residual cathepsin D activity with predominance of pre-cathepsin D protein, indicating impaired trafficking of this enzyme in the endolysosomal system. These phenotypes have been previously associated with decreased levels of Rbsn-5 achieved through siRNA silencing [5,17]. Understanding how the G425R mutation phenocopies Rbsn-5 depletion will require further structure-function studies outside the scope of the present findings.

The mechanisms by which deficiency of Rbsn-5 at the cellular level lead to the complex features of this patient are suggested by cases in which specific defects in the endosomal/lysosomal pathway produce complex clinical phenotypes. For example, primary cathepsin D deficiency [12,18], causes early onset epileptic encephalopathy and microcephaly, features, which are prominent in our patient’s clinical phenotype. Similarly, mucolipidosis type III, a lysosomal trafficking disorder caused by loss of mannose-6-phosphate targeting signals on lysosomal proteins, results in dysmorphic facial features, small stature and dysostotic bone changes similar to those observed in our patient [19,20]. The electron-dense material contained in the lysosomes of this patient’s fibroblasts may indicate lysosomal storage due to reduced intralysosomal amounts of enzymes dependent on mannose-6-phosphate mediated trafficking [19,20].

Alterations in endocytic trafficking caused by Rbsn-5 deficiency might be the common denominator for the pleiotropic clinical manifestations observed in this patient. For example, the severe dyslipidemia developed in response to ketogenic diet may in part be attributable to impaired LDL receptor trafficking. While the child’s initial vitamin B12 deficiency might in part be of nutritional origin, in combination with the observed albuminuria, impaired endocytic trafficking in proximal renal tubular cells, where transcobalamin and albumin are reabsorbed by the Megalin/cubilin receptor protein [21], might have been an aggravating factor. Likewise, the urine methylmalonic acid excretion and the red blood cell macrocytosis, despite cobalamin supplementation, may be due to defective intracellular trafficking of cobalamin. Although not entirely explained, the disproportionate synthesis of methylcobalmin and adenosylcobalamin in the patient’s fibroblasts might be an indication of this proposed mechanism as well.

There is also a possible relation between the observed bone marrow changes and Rbsn-5 deficiency. Missense mutations leading to depletion of Vps45, a known Rbsn-5 binding partner, have recently been reported in patients with severe neutropenia, bone marrow fibrosis and early lethality [22,23]. The reticular fibrosis found in our patient’s bone marrow resembles these findings. Functional folate deficiency caused by folate antagonistic anti-epileptic drugs (e.g. lamotrigine) and the KD also could explain these findings, although hematological studies proving such side effects are lacking.

The cumulative effects of Rbsn-5 deficiency on cellular function are manifested by a slow growth rate of the patients fibroblasts, compared to age matched controls, as well as by morphological abnormalities such as the accumulation of translucent vacuoles and intermediate filaments. The extent to which these abnormalities are directly attributable to Rbsn-5 function, or are secondary to chronic impairment of endocytic trafficking is unclear, but the failure of Rbsn-5 wild-type transfection to revert vacuolar phenotype is suggestive of the latter possibility. Nevertheless, impaired cytoskeletal dynamics have been seen in models of Rbsn-5 deficiency [24], and could explain the accumulation of intermediate filaments in the perinuclear cytoplasm of our patient. While changes in neurotransmitter recycling [25-27], cytoskeletal dynamics [28], or dendritic branching [29] are possible causes, the precise mechanism by which this mutation contributes to the pathogenesis of epilepsy, a key feature in this patient, remains to be determined.

## Conclusions

Overall, based on the critical role of Rbsn-5 in endocytic pathways, mutations affecting its functions are likely the basis of multi systemic disorders such those presented here. More patients with Rbsn-5 deficiency need to be identified in order to delineate the specific clinical and biochemical phenotype of this gene defect. Patients with epilepsy, growth retardation, dysmorphic features, multi-system involvement (bone abnormalities, myelodyplasia, albuminuria) and unexplained biochemical findings such as cobalamin deficiency and partial cathepsin D deficiency should be considered at risk.
